# Land-change dynamics and ecosystem service trends across the central high-Andean Puna

**DOI:** 10.1038/s41598-019-46205-9

**Published:** 2019-07-04

**Authors:** Santiago Madrigal-Martínez, José Luis Miralles i García

**Affiliations:** 1 Universidad Politécnica de Valencia, Camí de Vera, s/n, 46022, Valencia, Spain; 2 Universidad Ricardo Palma, Av. Benavides 5440, Santiago de Surco, Lima Peru; 3 Universidad Nacional Agraria La Molina, Av. La Molina s/n, La Molina, Lima Peru

**Keywords:** Ecosystem services, Socioeconomic scenarios

## Abstract

Mountain landscapes provide multiple ecosystem services that are continually vulnerable to land-change. These complex variations over space and time need to be clustered and explained to develop efficient and sustainable land management processes. We completed a spatiotemporal analysis that describes how different patterns of 6 land-change dynamics impact on the supply of 7 ecosystem services over a period of 13 years and across 25 provinces in the central high-Andean Puna of Peru. The appraisal describes: (1) how clusters of land-change dynamics are linked to ecosystem service bundles; (2) which are the dominant land-change dynamics that influence changes in ecosystem service bundles and (3) how multiple ecosystem service provision and relationships vary over space and time. Our analysis addressed agricultural intensification, agricultural de-intensification, natural processes and deforestation as the most critical land-change dynamics across the central high-Andean region over time. Our results show that most of the provinces were mainly described by a small set of land-change dynamics that configured four types of ecosystem service bundles. Moreover, our study demonstrated that different patterns of land-change dynamics can have the same influence on the ecosystem service bundle development, and transformation of large areas are not necessarily equivalent to high variations in ecosystem service supply. Overall, this study provides an approach to facilitate the incorporation of ES at multiple scales allowing an easy interpretation of the region development that can contribute to land management actions and policy decisions.

## Introduction

Half of the world population depends on mountain ecosystem resources that are continually vulnerable to land-change^1^, mainly determined by the consequences of human activities^2^ and by natural processes. Deforestation, agricultural intensification, agricultural de-intensification and urbanization are complex land-change dynamics documented in the high-Andean region^3–6^, yet, in-depth multi-temporal change approaches are required^7^. Understanding this complexity can help to implement land management processes to balance biodiversity conservation with human needs^8^ and also to measure the changes produced in the supply of ecosystem services^9,10^.

Ecosystem service (hereafter ES) concept, the human well-being obtained from nature^1^, has become an important integrated framework in sustainability science^11^. The ES framework facilitates ecosystem conservation opportunities^12^ and affords innovative and valuable data to help decision-making^13^. In this context, land use/land cover (hereafter LULC) models provide a high performance for explaining the provision of individual ES^14^, even so limitations are found predicting cultural and some regulating services^15^. Evaluation of ES using LULC maps and expert estimation is worldwide extended^16^, but scarce samples are found in mountain regions (e.g.^17,18^) and none in the phytoregion of moist Puna. The expert knowledge serves as a surrogate of empirical observations in many scientific researches^19^. Roche and Campagne^20^ have proved that expert knowledge through the matrix approach can be as valid as the use of empirical data or biophysical indicators for ecosystem service assessment. Moreover, when the source data is scarce, as in the study region^7^, this method can be the best accessible alternative for ecosystem service estimations^21^ and solve the necessity of more ES appraisals in highland territories^22^.

However, mountain landscapes provide multiple ES that varies over space and time manifested by several land-change dynamics, making necessary a spatiotemporal analysis to advance the knowledge of ES trajectories^23–25^, likewise the positive or negative interactions between them, namely synergies or trade-offs respectively^26^. This complex ecological state, of multiple ES linked to land use in change tendencies, is clarified with ES bundles^27^. Bundles of ES, sets of ES co-occurring with human activities across a landscape over time^28^, contribute to incorporate ES models into land use planning^27,29^. Nevertheless, predictive variables need to be assessed to complete ES bundles performance^15,30^. At present, there are no studies of ES bundles in the high-Andean region^30^ linking clusters of land-change dynamics with bundles of ES trends to be used as a framework for improving stakeholder decisions in land planning.

**Figure 1:**
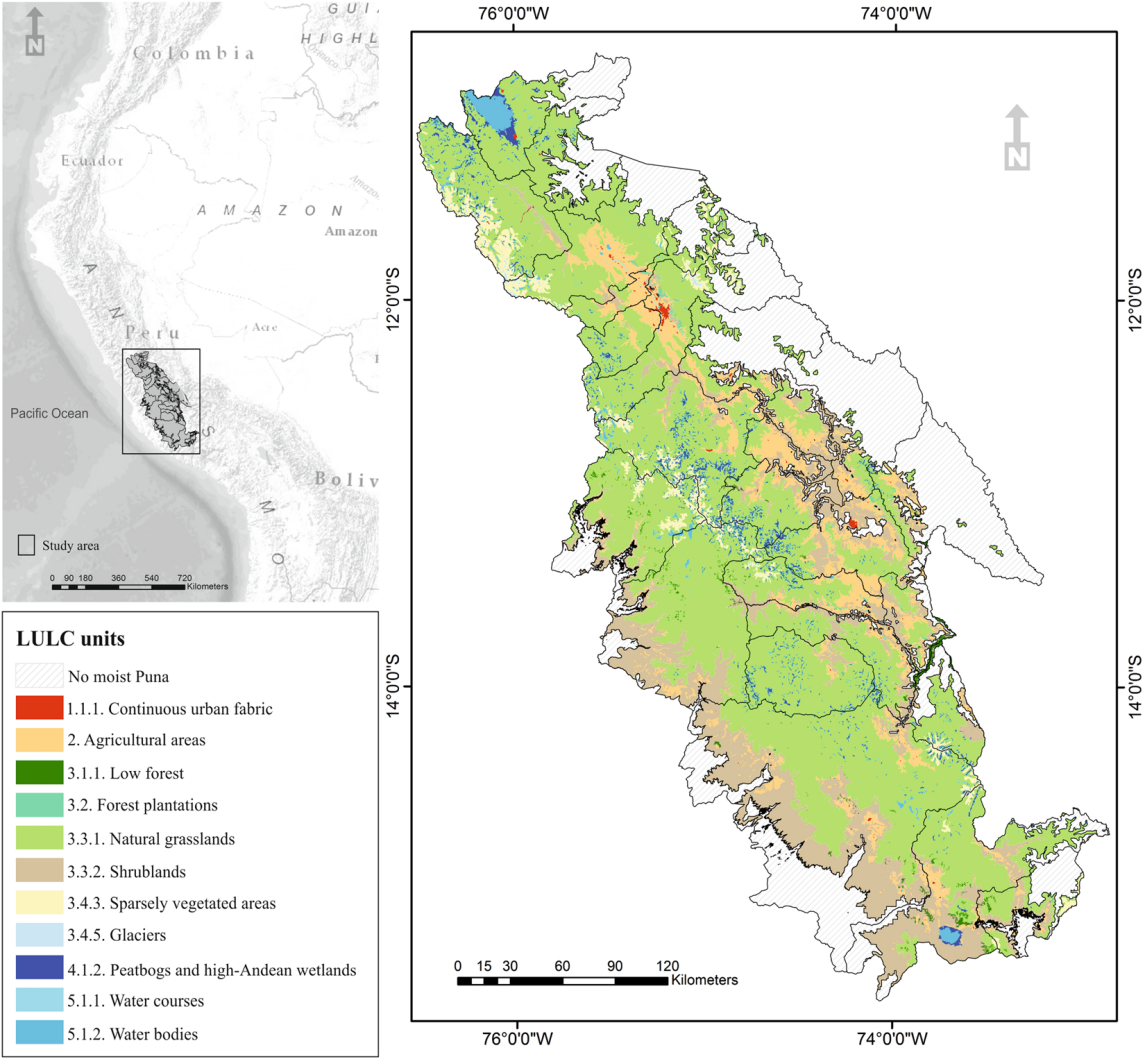
Study area (left) and relevant LULC units for the 25 administrative boundaries in 2013 (right). Maps were made using ArcMap 10.3 (http://desktop.arcgis.com/en/arcmap/). Study area background from World Reference Overlay (Sources: Esri, Garmin, USGS, NPS) and World Terrain Base (Sources: Esri, USGS, NOAA). Source administrative limits: Instituto Nacional de Estadística e Informática (Peru), http://geoservidorperu.minam.gob.pe/geoservidor/download.aspx. LULC map in 2013 made from official flora cover map, http://geoservidor.minam.gob.pe/recursos/intercambio-de-datos/.

Therefore, we develop a spatiotemporal analysis that describes how different patterns of 6 land-change dynamics impact on the supply of 7 ecosystem services over time (from 2000 to 2013) and across 25 provinces in the central high-Andean Puna of Peru (Fig. 1 left). We select this section of the moist Puna region (64,025 km^2^) as our study area because of its ecological significance and data availability. Moist Puna has an ecological importance as a sequester of great amounts of soil organic carbon, regulation of water flow and provision of farming outputs^31^. The 6 land-change dynamics represent the transitions assessed (Table S1) between the eleven relevant LULC units (Fig. 1 right) in the study period. The 7 selected ES include site-specific services from two main categories (five regulating and two provisioning) identified by the Common International Classification of Ecosystem Services^32^ and Burkhard *et al*.^33^: two regulating services related to mediation of flows (regulation of soil erosion and water flow regulation); one ES related to filtration, sequestration, storage or accumulation by ecosystems (water purification); two services linked to the maintenance of physical, chemical, biological conditions (soil quality and global climate regulation) and, finally, two provisioning services related to nutrition (crops and reared animals). First, we consult 63 practitioners in order to estimate the maximum capacity of each LULC unit to supply each of the regulating ES and we complete the potential supply of provisioning services from official model results. Second, we incorporate time in the spatial analysis to assess the land-change dynamics as achieved by other studies (e.g.^24,25^). Third, we investigate the associations between clusters of land-change dynamics and ES bundles, to identify positive, negative or contrasting patterns. Fourth, we determine the explanatory variables (e.g.^23^) that best predict these associations.

Our work provides a comprehensive view of how clusters of land-change dynamics are linked to ES bundles, and the social-ecological determinants (firewood, population, alpaca and distance from Lima) that explain these associations. We hypothesize that higher rates of LULC changes hardly modify ES supply and configuration of these changes had a critical role in ES bundle development; but our findings show that transformation of large landscapes are not necessarily equivalent to high variations in ES, whereas small land alterations are corresponding to slight impacts in ES. Our study highlights agricultural intensification, agricultural de-intensification, natural processes and deforestation as the most significant land-change dynamics that influence variations in ES bundles. We confirm that multiple ES provision and relationships vary over space and time. We hope our study will provide information that might promote and facilitate the incorporation of ES at multiple scales for sustainable land management.

## Results

### Ecosystem service matrix scores and sensitivity analysis

**Figure 2:**
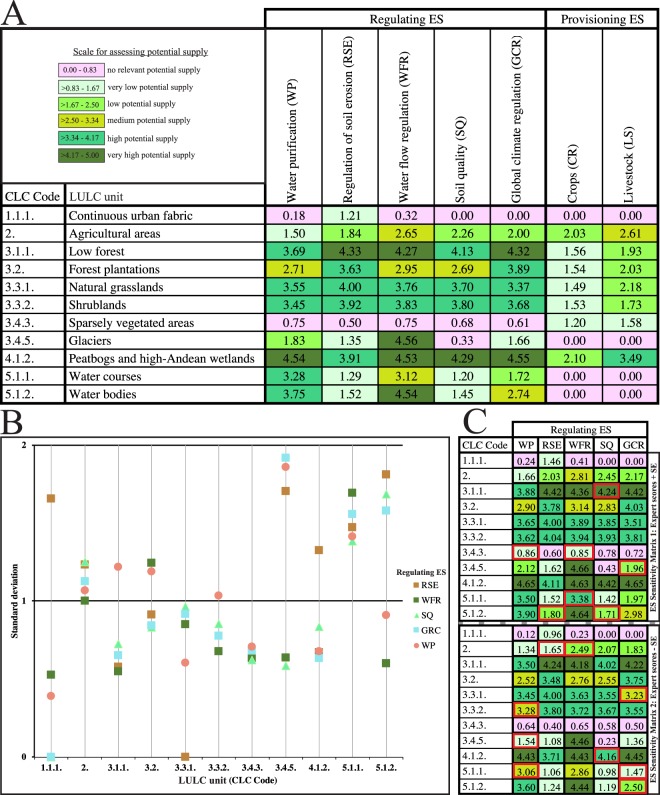
ES matrix (**A**) and descriptive statistics for the sensitivity analysis (**B**,**C**). (**A**) The matrix illustrates the flow of regulating and provisioning ES potential supply in the moist Puna. (**B**) The graph displays the standard deviation for expert responses in each LULC/regulating ES pairs. (**C**) The ES sensitivity matrix 1 shows the expert scores plus the standard error. The ES sensitivity matrix 2 presents the expert scores minus the standard error. The cells with red outline denote a one-level class variation in the potential supply.

The expert scores for regulating ES and the results of the standardised method for provisioning ES are presented in Fig. 2A. The details of the quantity of consulting experts, the outliers identified and the contributing answers for each LULC/regulating ES pairs are systematized in Resource 1.

Peatbogs and high-Andean wetlands (2.46% of the study area in 2013) afforded the highest potential for both ES sections. Low forest, natural grasslands and shrublands gave higher values for regulating ES. These classes covered separately 0.58%, 61.39% and 20.05% of the territory in 2013. Glaciers and water bodies had very high potential regulating water flow. Water bodies and water courses got high performance purifying water, whereas forest plantations highlighted by its soil erosion control and carbon sequestration. Agricultural areas (10.10% of the study area in 2013) presented low and medium potential for crops and livestock services, respectively. Finally, continuous urban fabric and sparsely vegetated areas are related with no relevance supply in almost all the ES.

The sensitivity analysis was carried out to evaluate the variability and the uncertainty in the regulating ES matrix scores. The variability of the expert responses had a low significance, varying between SD = 0 for agreements and up to SD = 1.918 for the biggest discrepancies (Fig. 2B). The results showed that 5% of the scores got an unanimous response, while 55% had very low variability. Glaciers and water bodies gathered the higher SD values with global climate change and regulation of soil erosion services, respectively. Although, water purification was the service that accumulated more percentage of discrepancies (11%), showing low reliability. Whereas, water flow regulation and soil quality services grouped 15% of low variability responses.

The comparison between the sensitivity matrices 1 and 2 (Fig. 2C) and the regulating ES matrix indicated 87% and 84% of overall agreement of cells under equal class of the potential supply, respectively. The minor differences supposed an increment or decrement one level in the potential supply scale in 7 and 9 expert scores after adding or deducting the SE value as it should. Kappa coefficient for the sensitivity matrices 1 and 2 were 0.84 and 0.79 representing “almost perfect” and “substantial” accuracy. By LULC, continuous urban fabric and forest plantation continued undisturbed after submitting the changes. Sparsely vegetated areas and water bodies have the largest potential increment, while agricultural areas and water courses show the biggest supplying reduction. By regulating ES, water flow regulation and soil quality services were the most upgraded, quite the opposite occurred with water purification and global climate regulation services. In summary, the low variability of the responses and stability around the mean values signified robustness of the regulating ES matrix scores for the studied area.

### Quantification of individual LULC changes and clusters of land-change dynamics

The details of LULC changes between 2000 and 2013 are presented in Tables S1 and S2 (Resources 1). In terms of the absolute area, 5676 km^2^ (8.8%) and 5461 km^2^ (8.5%) were transformed during the two time steps, respectively. The annual land-change for the first time period (T1) was 630.7 km^2^ per year, whereas for the second time step (T2) was 1365.3 km^2^ per year. Kappa analyses confirmed a strength of agreement of “almost perfect” among the two time step maps.

Natural grasslands coincided to be the largest class in each year (above 60%) seconded for shrublands and agricultural areas, configuring the 90% of the study landscape. However, twenty-two (T1) and twenty-four (T2) types of transitions were assessed and grouped in six land-change dynamics (Table 1). Agricultural expansion (D1) was the more extensive land-change dynamic in T1, implicating the conversion of low forest, shrublands and natural grasslands. Agricultural de-intensification (D2) represented an increase of grasslands and shrublands due to fallowing and/or land abandonment, largely registered during the second time step. Deforestation (D3) of low forest gave way to shrublands and natural grasslands, increased during T1 and decreased during T2. Dynamic type 4 represented by urbanization showed that urban areas slightly augmented by the encroachment of natural grasslands and agricultural areas. Afforestation (D5) of pine and eucalyptus species had a higher increase during the first time step, whereas in the second time period showed a slight growth. Natural processes, land-change dynamic type 6, set diverse type of changes during T1, highlighting the reduction of nival zones (−66.78%), boosting the expansion of sparsely vegetated areas. Concerning T2, there were important transitions registered as the extensive reduction of peatbogs and high-Andean wetlands increasing natural grasslands.

**Table 1:** Estimated area (km^2^) of each type of changes and land-change dynamics occurred from 2000 to 2013 in the study area.

Code	Land-change dynamic	Type of change	2000–2009 (T1)		2009–2013 (T2)	
			km^2^	%	km^2^	%
D1	Agricultural expansion	LF to AA	16.3	0.3	8.1	0.15
		SHL to AA	556	9.8	133.2	2.44
		NG to AA	2400	42.3	542	9.92
		PWL to AA	0	0	24.9	0.46
D2	Agricultural de-intensification	AA to NG	233.6	4.1	599.4	11
		AA to SHL	0	0.0	1492.7	27.3
D3	Deforestation	LF to NG	2.1	0.04	168	3.1
		LF to SHL	1068.8	18.8	157.4	2.8
D4	Urbanization	NG to CUF	0	0	3.8	0.1
		AA to CUF	0	0	7.6	0.1
D5	Afforestation	NG to FP	96.7	1.7	10	0.2
		AA to FP	4.1	0.1	0	0
		SHL to FP	10.5	0.2	0	0
D6	Natural processes	Miscellaneous	1288	22.7	2313.8	42.4
Total			5676	100	5461	100

LULC classes and abbreviations: Continuous urban fabric (CUF), Agricultural areas (AA), Low forest (LF), Forest plantations (FP), Natural grasslands (NG), Shrublands (SHL) and Water courses (WC).

**Figure 3:**
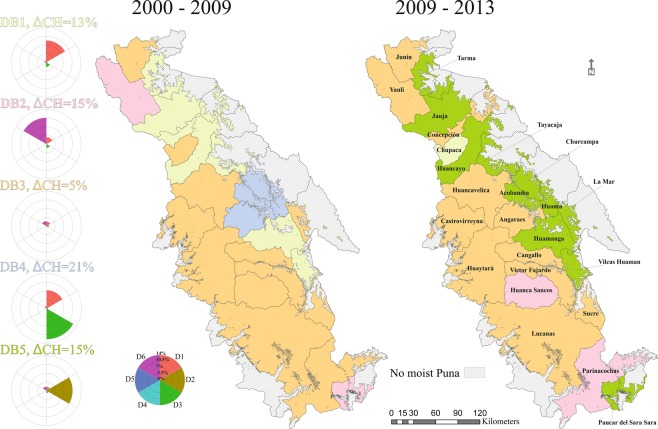
Clusters of land-change dynamics spatially distributed over the two-time periods. Star plots illustrate the land-change dynamics and the total percentage of transformed land (∆CH) for each cluster. Each ray length is proportional to the percentage of changed land of its corresponding dynamic (rays are comparable within clusters). Dynamic types and abbreviations: agricultural expansion (D1), agricultural de-intensification (D2), deforestation (D3), urbanization (D4), afforestation (D5) and natural processes (D6).

The provinces were grouped into five types of clusters based on the kind and proportion of land-change dynamics occurred through time (Fig. 3). The bundle type 1 (∆CH = 13%), characterized eight provinces (seven in T1 and one in T2) with a dominant process of agricultural expansion following by a slight reduction of low forest. Two provinces in each time period (cluster DB2, ∆CH = 15%) were mainly controlled for natural processes, highlighting glaciers retreat (during T1) and reduction of peatbogs and high-Andean wetlands in the final period. The third bunch (DB3) considered the provinces practically undisturbed (12 provinces for 2000–2009 and 11 provinces for 2009–2013). Whereas, group type 4 (DB4), displayed four provinces that experienced the biggest LULC changes (∆CH = 21%), due to deforestation and agricultural expansion, during the initial time period. The fifth bundle (DB5, ∆CH = 15%) defined eleven provinces by their agricultural de-intensification in the final time period. It should be noted that urbanization (D5) and afforestation (D6) had very short percentage of changed land, graphically imperceptible in each star plot (Fig. 3).

DB3 (lowest land-change trend) is the dominant cluster in the two-time periods, characterizing 48% and 44% of provinces respectively. From this group, eight provinces (32%) kept unalterable trends through time. Despite this uniformity, there were nine different changes followed by the provinces (Fig. S1A Resource 1). Three principal types of variations described the 65% of all the changes. Six DB1 and three DB4 provinces changed to become DB5 showing a clear trajectory of agricultural abandonment. Two provinces DB3 (Parinacochas and Huanca Sancos) changed to DB2 due to enlargement of shrublands and drying of peatbogs and high-Andean wetlands correspondingly.

### Bundles of ES trends and relationships among individual ES trends

**Figure 4:**
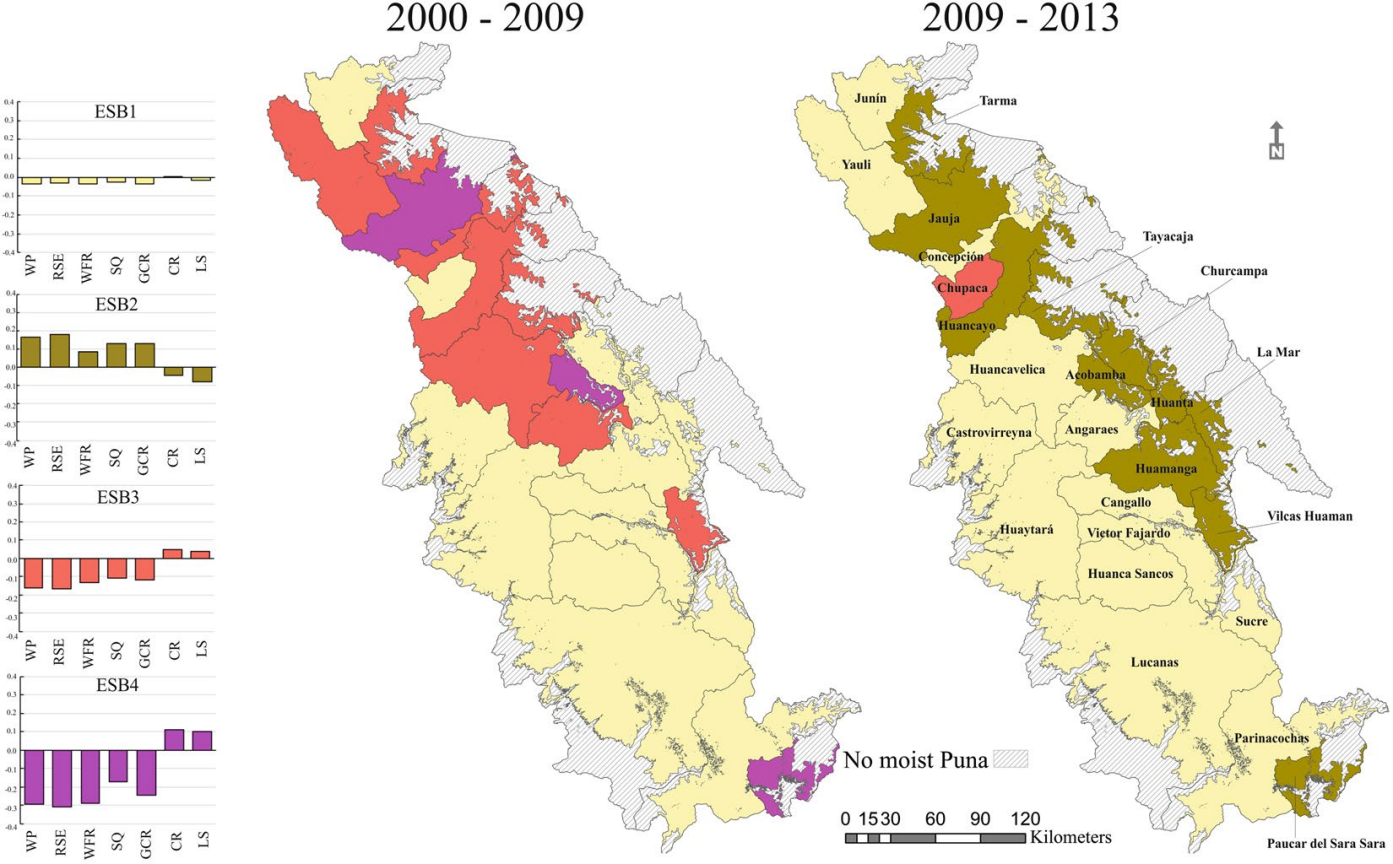
Spatial distribution of ecosystem service bundles (ESB) grouping the ES potential average trends over the two-time periods. Barplots show the ES potential average variation within each bundle type. Ecosystem service types and abbreviations: water purification (WP), regulation of soil erosion (RSE), water flow regulation (WFR), soil quality (SQ), global climate regulation (GCR), crops (CR) and livestock (LS).

Cluster analysis defined four groups based on ES potential average trends of each province boundary over time (Fig. 4). The bundle type 1, ESB1 revealed that twenty-seven provinces (fourteen in T1 and thirteen in T2) had a slight loss in regulating services and a constant supply of provisioning services over time. Eleven provinces (Bundle ESB2) experienced an improvement of regulating services and a reduction of provisioning in the final time period. The positive changes occurred under a trend of land abandonment and fallowing. Bundle ESB3 showed provinces (primarily in T1) with an overall change that had negative effects on regulating services. The fourth bundle (ESB4) characterised three provinces that enlarged their potential of provisioning services and highly reduced regulating services.

Nine ESB1 provinces formed a large cluster with low variability in ES provision reflecting low changes in the landscape through time. Sixteen provinces changed their bundles over time defining mainly four different paths (Fig. S1B Resource 1). Thirty percent of provinces providing ESB1 (low trend of ES supply) in T1 changed to ESB2 (increasing trend of regulating ES and decreasing trend of provisioning ES) by the final time period, reflecting a tendency of agricultural abandonment. Provinces characterised by a strong negative trend in regulating services (ESB4) in the initial time period changed to ESB2 in T2, showing the recovery of ecosystems. Ninety percent of ESB3 provinces changed equally to ESB2 or ESB1 by T2, displaying a landscape with a positive trend in regulating ES. Only one province (Chupaca) increased provisioning services supply (ESB3) as a detriment of regulating ES.

At phytoregion scale, the type and strength of the interactions among ES trends over the two-time periods are detailed in Table S3 (Resource 1). Regulating services correlations were strongly positive through time. Trade-offs appeared with high strength among provisioning and regulating services for both time periods, only soil quality had a not significant negative relationship with livestock during the first time step. Crops and livestock services had a strong positive correlation through time. Twenty interactions for initial time period were significantly (p < 0.05), whereas each interaction for T2 were significant.

### Associations between clusters of land-change dynamics and ecosystem service trends

**Figure 5:**
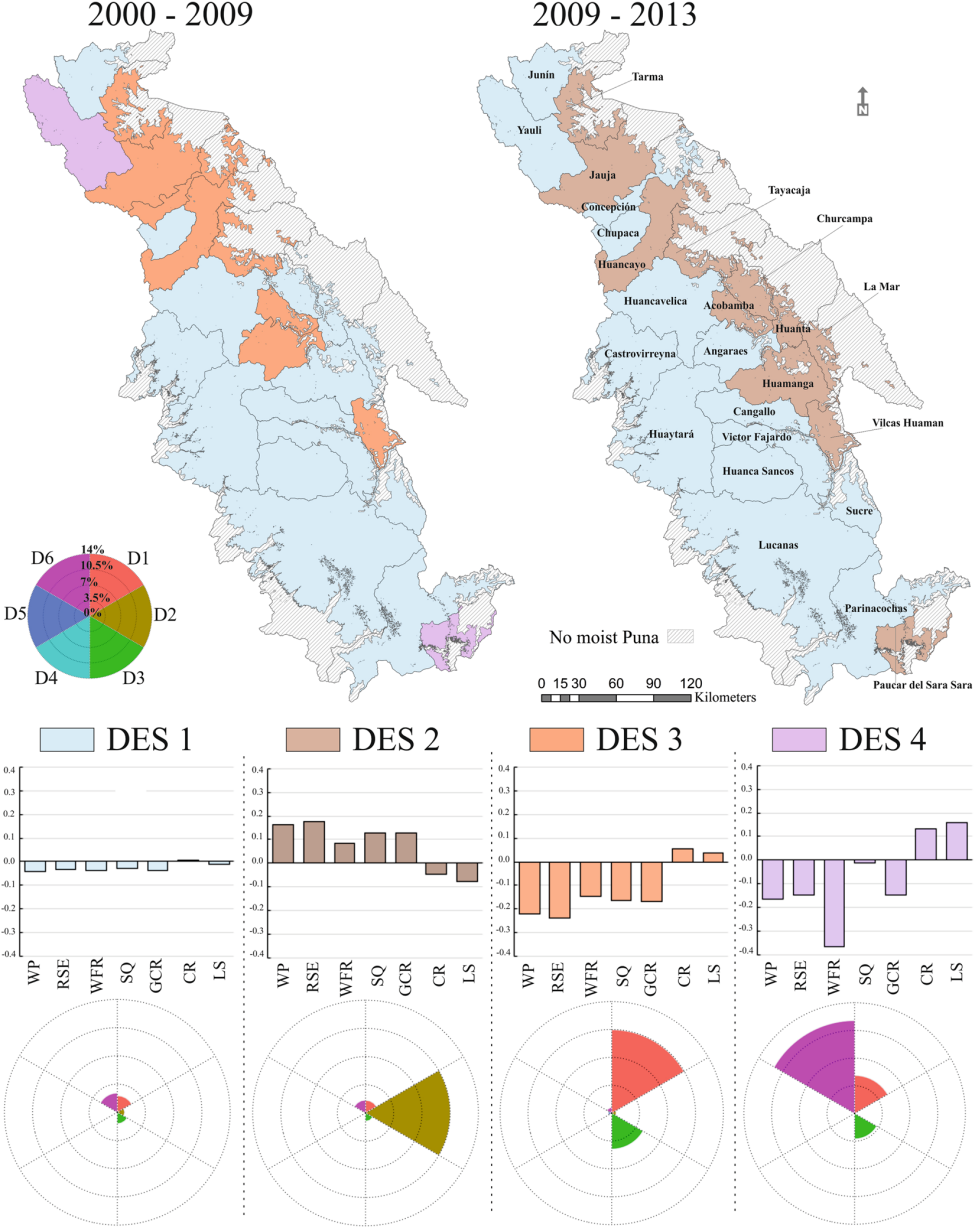
Spatial distribution of links over the two-time periods. Star plot and barplot describes each link between clusters of land-change dynamics and ecosystem service trends. Star plots illustrate the land-change dynamics and the total percentage of transformed land occurred in each cluster. Each ray length is proportional to the percentage of changed land of its corresponding dynamic (rays are comparable within clusters). Barplots show the ES potential variation within each bundle. Ecosystem service types and abbreviations: water purification (WP), regulation of soil erosion (RSE), water flow regulation (WFR), soil quality (SQ), global climate regulation (GCR), crops (CR) and livestock (LS). Dynamic types and abbreviations: agricultural expansion (D1), agricultural de-intensification (D2), deforestation (D3), urbanization (D4), afforestation (D5) and natural processes (D6).

Overlap and cluster analysis defined four links between land-change dynamics and ES trends (Fig. 5). The first link (DES1, ∆CH = 7%) is the largest in both time periods, grouping 30% and 28% of provinces respectively, mainly connecting ESB1 and DB3 clusters (80% of the connections in the group). This cluster showed a territory with a slight decrease in regulating services and minor variation of provisioning services, including provinces (Junin, Huaytara and Castrovirreyna) with a land-change proportion lower than 3% for both time periods. However, there were two provinces in T1 (Huanta and Churcampa, association ESB1 and DB4) with higher change proportion (12% and 19%) dominated by deforestation (70% of the strength for both provinces). Also, one province ESB1 and DB1 (Huamanga, ∆CH = 14%) was marked by a growth of farming and deforestation in T1.

Group DES2 (clusters DB5 with ESB2) defined eleven provinces in the final-time period (44% of the territory) with 15% of transforming land, characterizing areas by agricultural de-intensification (71% of the strength), that increased regulating services supply and decreased provisioning ES. In this link the two provinces (Huanta and Churcampa) that gathered the highest land-change proportion (23% and 22% respectively) also experienced a severe deforestation process (42 and 50 km^2^ correspondingly).

The third link (DES3) is composed principally by DB1 and ESB3 provinces, describing eight provinces in the initial-time period that produced a high land-change proportion (∆CH = 17%), primarily due to agricultural intensification and deforestation (60% and 27% of the total average change calculated by this link respectively). These changes produced positive effects on provisioning services at the expense of regulating ES. It should be noted that a province (Acobamba, ESB4 and DB4) had the largest individual land-change (36%), resulting in 15% of agriculture extension and 19% of forest decline in its territory.

Two provinces formed the fourth association (DES4) characterised by a positive supply of provisioning services and negative trend of regulating ES (ESB3 and ESB4) obtained with a land-change average of 20% during the first-time period. Both provinces are determined by bundle DB2 highly induced by natural processes (60% of the total average change calculated by this link), that affected negatively water flow regulation. It should be noted that increase of crops and livestock potential were as a consequence of glaciers retreat and expanding agricultural frontier.

The spatial distribution of associations between clusters of land-change dynamics and ecosystem service trends changed through time. Although DES1 (slight land-changes and minor ES variations) was the dominant link in both time periods, making a large group of eleven provinces, there were three relevant variations followed by the remaining fourteen provinces (Fig. S1C Resource 1). Six provinces defined by link DES3 (crops and livestock expansion), one DES4 province (crops, livestock and water flow regulation fall) and four DES1 provinces changed to DES2 (regulating services), reflecting tendencies toward crop production specialization following agricultural de-intensification. Two provinces DES3 and one province DES4 also changed to enlarge DES1 cluster.

At regional scale, the development occurred in the initial-time period displayed a territory influenced by land-change dynamics that caused an improvement of crops and livestock provision, largely due to agricultural intensification. This condition, together with natural processes and deforestation generated negative effects on regulating service provision. Whereas, the final-time period modelled a landscape with a positive tendency of regulating ES, where land abandonment was the dominant land-change dynamic.

The redundancy analysis (RDA) revealed the important land-change dynamics for predicting the variability of ES within each province over the two time periods. Both land-change models had a high capacity to explain the performance of ES (Model T1: R^2^ = 0.949 and P-value < 0.001; Model T2: R^2^ = 0.952 and P-value < 0.001). In order to their partial contribution, the significant dynamics for model T1 were agricultural intensification, natural processes, deforestation and agricultural de-intensification. Whereas for model T2 were agricultural de-intensification, agricultural intensification and natural processes. Afforestation and urbanization had insignificant influence in the distribution of individual services in both models, whereas deforestation was irrelevant for model T2. Results of RDA analysis are in Table S4 in Resource 1.

### Determinants for dynamics and ecosystem services

The RDA specified firewood, population, alpaca and distance from Lima as the relevant variables that finest explicated the two-time models generated by land-change dynamics and ES trends, R^2^ = 0.36 and P-value < 0.001. Each explanatory variable displayed different spatial distribution within the study area (Fig. S2 in Resource 1). Firewood consumption showed higher values in the initial time period in all the provinces. In contrast, the density of alpacas presented an increment in almost each province during the second-time period. Population density varied slightly between both periods, characterising a territory with eleven provinces in a growing rate and fifteen provinces with a declining proportion over time. Distance from Lima showed that most of the provinces are situated beyond four hundred kilometres.

Figure S3 (Resource 1) plot (scaling 2) the RDA results for land-change dynamics and ES trends across the moist Puna. Most of the provinces with very low changes in ES provision and land (DES1) were remote from Lima, had a low population density, a growing alpaca activity and low firewood consumption. Provinces that experience an increase of regulating services and a reduction of provisioning (DES2) during the final time period were related to areas with low alpaca density, high population density and middle-low distance to Lima. Provinces with an augmentation of provisioning services (DES3) and reduction in regulation services during the first-time period stayed in areas with high population density and growing fuel wood needs. The two provinces (DES4) during the initial time period had medium consume of firewood, high expansion of alpaca breeding and low-medium population density.

## Discussion

The involvement of 63 national and international experts with recognized experience developing ecological studies in the research field and being free to fulfil only the well-known LULC/regulating ES connections increased the confidence. The starting list of experts was short and grew by their suggestions as a “snow ball” sampling technique^34^, taking the example by Scolozzi *et al*.^35^. Nevertheless, the final respondent pool was carefully selected from the larger number of qualified references following the indications by Jacobs *et al*.^16^. This strategy assured a high rate of participation (68%, 43 experts were interviewed) in a low period (07 weeks). Finally, after removing outliers, an average of 39 interventions was computed getting low variability in the final scores and reaching a stable mean, in concordance with Campagne *et al*.^36^, and validated by the results of the sensitivity analysis.

Expert favourably scored low forest and peatbogs and high-Andean wetlands, in a certain way expressing comparable opinions with specialists from around the world^33,37,38^. On the contrary, urban zones were scored as low as possible for many of the experts, coinciding with results from matrix model international studies^18,33,39,40^. Agricultural areas got medium-low potential supply showing similar analyses pointed, in other studies^41,42^. Glaciers and water bodies were highlighted as water flow controllers matching scores from Burkhard *et al*.^33^. Forest plantations had medium-high attention from experts, these scores were slightly higher from the ones expressed by Montoya-Tangarife *et al*.^38^ with identical species. Natural grasslands and shrublands develop important functions in the study area by their nature and spatial magnitude, as concerned by the practised.

At regional scale, the ES matrix captured a landscape with a richness in regulating services differing from the scores of provisioning services, that according to official studies, presented a region with medium-low potential for crops and livestock.

Cluster analysis for LULC changes confirmed that most of the provinces were mainly described by a small set of dynamics, but with one dominant force. Only one bundle that included the biggest LULC changes (DB4) was rather specialized in two dynamics. Three clusters were characterised by human actions and one by natural processes, just the bundle with the lowest ratio of change (DB3) had a quite diverse combination of forces. Urbanization and afforestation affected the lowest number of zones. Land-change dynamics described in the clusters are consistent with the stated in other regional studies^3,43–46^.

Change over time analysis in pairwise interactions among ES described a strong significant correlation, revealing trade-offs among provisioning and regulating services; and synergies concerning the same ES sector. At similar landscapes, livestock trade-off global climate regulation, water flow regulation^47^ and regulation of soil erosion^48^. Turner *et al*.^49^ assessed a strong relationship between provisioning services (crops and livestock) and negative interaction with water purification. In an agricultural landscape, a pattern of trade-offs was found between provisioning and regulating ecosystem services^28^. Agudelo-Patiño and Miralles-Garcia^50^ indicated that provision of crops compromised water flow regulation in an Andean urban mountain system.

ES bundles showed four different trends that linked the five land-change clusters establishing four types of associations. Provinces DSE1 were located in both time periods covering 72% and 69% percent of the territory, respectively. Although this landscape was the less undisturbed (∆CH = 7%), accumulated the 38% of deforestation (during first-time period), 35% of agricultural expansion (in both time periods) and 85% of urbanization (during T2). Urbanization has negative effects on water infiltration^50^ initiating surface run-off^51^ and losses of carbon stocks and crops^52^.

Link DES2 displayed eleven provinces that increased regulating ES potential due to an important process of agricultural de-intensification in T2 (79% of the total change caused by this dynamic in the study area over 13 y). Farming reduction co-occurred with a very low intensity of deforestation and a small increase of farming land (10% and 12% of the total change caused by each dynamic in the study area, respectively). The abandonment of marginal agricultural lands facilitates ecosystem recovery^5^. Loss of soil fertility indicates shrublands regeneration^53^. Evergreen vegetation regrows in natural fallow lands controlling soil erosion^54^. Abandoned pastures contribute to C-sequestration^55^.

The expansion of agriculture was the dominant dynamic in association DES3 occurred in the first-time step. Eight provinces had an enlargement of provisioning services and a high reduction of regulating ES (accumulated the 49%, 38% and 64% of the total change caused by agricultural expansion, deforestation and afforestation over the study period, respectively). It is proved that appropriate climatic conditions support crop development in higher elevation areas^56^ affecting natural grasslands that could reduce water flow regulation and livestock services^31^. Agricultural intensification reduce or eliminate fallow producing degradation of soils^57^.

Association DES4 (accumulated 20% of the total change caused by natural processes in the study area) described two provinces in the initial time period with high loss of water flow regulation primarily due to glaciers retreat. In Peru, loss in surface area of glaciers is manifested in the last two decades^58^ that may impact on water resources^59^ and arise land for grazing and farming^6^.

Local social-ecological determinants explained where changes in associations of land-change dynamics and ES trends occurred across the moist Puna. Provinces (DES1) characterised by low human-altered landscapes were quite inaccessible from Lima and with a very low population density, whereas landscapes dominated by agricultural intensification were associated with a growing population density and developed road network. Areas (DES2) distinguished by a rise of regulating services were associated with a reduction of fuel wood consumption, whereas provinces with high deforestation were related to an increase in firewood use. Provinces defined by a growth of ES provision (DES4) were correlated to a high promotion of alpaca breeding.

Our study focuses on cluster analysis over time on a provincial scale, since in Peru land planning at local level is regulated by provincial municipalities (Organic Law of Municipalities No. 27972, 27 of May of 2003). The integration of ES in planning depends on the governmental planning instruments^13^, therefore our study might promote and facilitate the incorporation of ES at multiple scales. Furthermore, in relation to the temporal scale of 13 years, the tendency of changes occurred as consequences of land management activities were observable in the territory. However, long historical data can improve the understanding of ES dynamics^23–25^, but in the study area, availability and quality of past LULC models are absent.

Overall, our analysis addressed agricultural intensification, agricultural de-intensification, natural processes and deforestation as the most critical land-change dynamics and their grouping across the high-Andean region through the 13 y. These clusters configured four types of ES bundles that might clarify ES complexity and help management purposes and decision-making.

The results have demonstrated that different patterns of land-change dynamics can have similar influence on the ES bundle development. The transformation of large areas are not necessarily equivalent to high variations in ES supply, whereas small land alterations are corresponding to slight impacts in ES provision. Moreover, trend mapping as expressed by Van Jaarsveld *et al*.^60^ is suitable for measuring modifications in ES supply, based on LULC differences^25^. Lastly, the approach grounded on an expert-based ES matrix emphasising the competence of the methodology in locations with data scarcity.

## Methods

### Study area

The study is focusing on the phytoregion of the moist Puna comprised within the administrative boundaries of 25 provinces in the departments of Junín, Huancavelica and Ayacucho (Fig. 1). The population at the end of 2017 was 2 096,156. Provincial area ranged from 724 to 10,999 km^2^ with an average of 2561 km^2^. Its geography is characterised by high plateaux and inter-Andean valleys (3500 m.a.s.l.) with a vegetation dominated for natural grasslands and shrublands^61^. Human interventions at work have been done during several millennia^43^ configuring agro-ecosystems based in an extensive livestock rearing and smallholdings of Andean crops^62^.

### Data set

The study quantified the changes on the provision of ES over the interval of 13 years, from 2000 to 2013, using a selection of LULC types included in the standardized nomenclature of the Corine Land Cover (CLC) for Peru. This nomenclature adapted from the European Commission CORINE programme is based on a 3-level hierarchical classification system comprising 43 land-cover classes at its most detailed level, 16 classes at level II and five classes at level I. Mainly, the spatial data set was derived from three sources, map of high-Andean ecosystems in 2000^61^, the official flora cover map from 2009^63^ and the official flora cover map from 2013^64^. These are polygon shapefiles generated in a mapping scale of 1:100,000 with Landsat (TM) images. Eleven relevant LULC were identified in the study area (Fig. 1), only one (agricultural areas) is a class I due to coarse attributes. Table S5 in Resource 1 presents the features of the three time data sources and their harmonization to extract the research LULC. Therefore, the “intersect” and “dissolve” tools in ArcGIS 10.3.^65^ were used to improve the integration of data for the three LULC maps in polygon shapefiles prepared for expert-based ES evaluation.

### Ecosystem services matrix

The ES matrix is an expert-based estimation technique^14^ that is extensively used to overcome data scarcity^37,38^. However, uncertainties are included in the scoring assessment^16,66^. In order to avoid this, Campagne *et al*.^36^ measured that 30 experts are enough to get a stable mean without inconsistencies and the variability of the final scores is constant after 15 experts, decreasing the standard error when increasing the expert panel size. For this study, 43 national and international experts (see respondent pool particulars in Resource 1), that have published scientific or technical works about ES or related ecological processes in the moist Puna, were individually consulted to rank the ES potential supply associated with a specific LULC on a relative scale, ranging from 0 (no relevant ES potential supply) up to 5 (very high ES potential supply). Burkhard *et al*.^39^ conceptualize the ES potential as the hypothetical maximum capacity of a LULC to supply a specific ES. Our matrix linked eleven LULC classes and seven ES, including regulating (n = 5) and provisioning (n = 2). To increase confidence, experts fulfilled only the LULC/regulating ES pairs that were surely in their judgments. Each response was collected and deprived of outliers using the interquartile range method (see Table S7, Resource 1). Then, a final score was computed using the mean. The potential supply of the LULC in provisioning services was achieved from official model results (see Table S8, Resource 1).

A sensitivity analysis was performed using descriptive statistics to prove the robustness of the regulating ES matrix. The standard deviation (SD) and the standard error (SE) were calculated from expert scores with the intention of ascertaining variability of the responses and uncertainty around the mean values, respectively. For variability control, given that match expert scores denote null SD, the answers were ranked in two categories, very low variability for SD ≤ 1 and low variability for SD higher than 1 and lower than 2. On the other hand, the uncertainty assessment was completed developing two sensitivity matrices with the expert scores ± SE (matrix 1 with expert scores + SE and matrix 2 with expert scores –SE). The kappa values were computed to obtain the degree of agreement between the ES regulating matrix and the sensitivity matrices.

### Assessing the changes for LULC dynamics and ecosystem services

Initially, LULC changes for 2000–2009 (T1) and 2009–2013 (T2) were detected, showing the quantity of land that was converted from each LULC to any other. Furthermore, the consistencies were evaluated with kappa statistics^67,68^. The transitions assessed were grouped in main dynamics and their proportion of change at province scale for the two-time periods were estimated with Excel 2015. To obtain the proportion of change for each province, the area difference of each land-change dynamic between final year to initial year was divided by the area of the province respectively as appropriate.

Then, ES trends for each province and time period were estimated using equation (1):

$${\rm{ES}}\,{{\rm{trend}}}_{{\rm{n}}}=[\frac{{\rm{\Sigma }}{({{\rm{A}}}_{{\rm{i}}}\ast {{\rm{ES}}}_{{\rm{i}}})}_{{\rm{t2}}}}{{{\rm{A}}}_{{\rm{n}}}}]-[\frac{{\rm{\Sigma }}{({{\rm{A}}}_{{\rm{i}}}\ast {{\rm{ES}}}_{{\rm{i}}})}_{{\rm{t1}}}}{{{\rm{A}}}_{{\rm{n}}}}]$$

where A_i_ is the area of LULC unit *i*, ES_i_ is the score assigned to the LULC unit *i*, A_n_ is the total study area of the province *n*, t_2_ means that the data correspond to final year and t_1_ means that the data correspond to the initial year of the time period.

Secondly, clusters of land-change dynamics (DB) and bundles of ES trends (ESB) were identified on the two-time periods. DB were delineated with the annual proportion of LULC change accounted for land-change dynamics in each administrative boundary. ESB were defined with the ES trends of each province boundary. The optimal number of partitions for both cluster models was found with “affinity propagation” method^69^ using R^70^. DB and ESB were mapped with ArcGIS 10.3^65^.

The relationships between individual pairs of ES (n = 21 pairs) through time were achieved with Spearman’s rho using the ES trend values for each time period. Significant correlation (p < 0.05) in negative relationships indicated trade-offs, whereas positive interactions were defined as synergies.

Thirdly, to assess the links between clusters of land-change dynamics and bundles of ES trends, the spatial correspondence between the models was assessed by overlap analysis. Then, we gathered the overlapped clusters according to the number of partitions obtained with “affinity propagation” method^69^ using R^70^.

Lastly, the land-change dynamics that best explained the variation of ES were determined using RDA (“vegan” R package and the function “ordistep^71^”).

### Identifying drivers for dynamics and ecosystem services

In our case study, RDA was computed for land-change dynamics and ES trends. The evaluation determined how land-change dynamics and ES trends were related to seven potential drivers (population, mining, alpacas, goats, firewood, distance from Lima and slope). These drivers were selected due to their role as explanatory variables used for dynamics or ES modelling. Deforestation in the moist Puna is related to anthropic actions like felling, firewood, fire and goat overgrazing^72^. Depopulation of rural zones explain agricultural abandonment^5^. Population growing increase town areas affecting many ecosystem services. Slope is negative relate to livestock and crops services^15^. Mining claims have consequences on Andean ecosystems and especially on water quality^73^. According to location theory the distance from an urban centre will define the activities for that territory.

RDA was calculated using the “vegan” R package and the function “ordistep^71^”, in order to obtain the best significant model (combination of explanatory variables), after 10,000 permutations^74^. Values of each driver were achieved for each period (T1 and T2).The data was obtained from census statistics, mining database and physiography model (the details of methods and data collection of drivers are in Table S9 Resources 1).

## Supplementary information

Online Resource 1

## Data Availability

All data generated or analysed during this study are included in this published article (and its Supplementary Information file).
