# Supplementary material for: Land-change dynamics and ecosystem service trends across the central high-Andean Puna
Source: Sci Rep. 2019 Jul 4;9:9688. doi: 10.1038/s41598-019-46205-9 (PMC6609706; doi:10.1038/s41598-019-46205-9)
Supplement: Supplementary file 1 — Online Resource 1 [file 41598_2019_46205_MOESM1_ESM.docx]

Supplementary Material

**Land-change dynamics and ecosystem service trends across the central high-Andean Puna**

Santiago Madrigal-Martínez^1,2,3*^ and José Luis Miralles i García^1^

^1^Polytechnic University of Valencia, Camí de Vera, s/n, 46022 Valencia, Spain

^2^Ricardo Palma University, Av. Benavides 5440, Santiago de Surco, Lima 33, Peru

^3^La Molina National Agrarian University, Av. La Molina s/n La Molina, Peru

*Author for correspondence: santiagomadrigal@lamolina.edu.pe

Table S1: Quantification of LULC transformations (in square kilometres) and change ratio occurred between 2000 and 2009 in the study Moist Puna

| 2009 | 1.1.1. | 2. | 3.1.1. | 3.2. | 3.3.1. | 3.3.2. | 3.4.3. | | | 3.4.5. | | 4.1.2. | | 5.1.1. | | 5.1.2. | | Total | | % | | Change  (%)* |
| --- | --- | --- | --- | --- | --- | --- | --- | --- | --- | --- | --- | --- | --- | --- | --- | --- | --- | --- | --- | --- | --- | --- |
| 2000 |  |  |  |  |  |  |  |  |  |  |  |  |  |  |  |  |  |  |  |  |  |  |
| 1.1.1. | 117.3 | 0.0 | 0.0 | 0.0 | 0.0 | 0.0 | | 0.0 | 0.0 | | 0.0 | | 0.1 | | 0.0 | | 117.5 | | 0.18 | | -0.05 | |
| 2. | 0.2 | 4881.4 | 16.3 | 0.0 | 2400.0 | 556.0 | | 0.0 | 0.0 | | 0.0 | | 0.0 | | 0.0 | | 7853.9 | | 12.27 | | 53.10 | |
| 3.1.1. | 0.0 | 10.6 | 677.5 | 0.0 | 4.4 | 10.0 | | 0.0 | 0.0 | | 0.0 | | 0.0 | | 0.2 | | 702.7 | | 1.10 | | -60.18 | |
| 3.2. | 0.0 | 4.1 | 0.2 | 41.7 | 96.7 | 10.5 | | 0.0 | 0.0 | | 0.0 | | 0.0 | | 0.0 | | 153.1 | | 0.24 | | 267.58 | |
| 3.3.1. | 0.0 | 233.6 | 2.1 | 0.0 | 38656.9 | 27.5 | | 0.0 | 41.8 | | 40.1 | | 0.0 | | 3.9 | | 39006.0 | | 60.92 | | -6.58 | |
| 3.3.2. | 0.0 | 0.1 | 1068.8 | 0.0 | 556.1 | 8737.9 | | 0.0 | 0.0 | | 0.0 | | 0.0 | | 0.0 | | 10362.8 | | 16.19 | | 10.90 | |
| 3.4.3. | 0.0 | 0.2 | 0.0 | 0.0 | 41.0 | 2.1 | | 1458.7 | 519.1 | | 1.6 | | 0.0 | | 0.0 | | 2022.7 | | 3.16 | | 38.66 | |
| 3.4.5. | 0.0 | 0.0 | 0.0 | 0.0 | 0.0 | 0.0 | | 0.0 | 282.9 | | 0.0 | | 0.0 | | 0.0 | | 282.9 | | 0.44 | | -66.78 | |
| 4.1.2. | 0.0 | 0.0 | 0.0 | 0.0 | 0.0 | 0.0 | | 0.0 | 0.0 | | 2652.3 | | 0.0 | | 0.0 | | 2652.3 | | 4.14 | | -2.31 | |
| 5.1.1. | 0.0 | 0.0 | 0.0 | 0.0 | 0.0 | 0.0 | | 0.0 | 0.0 | | 0.0 | | 67.8 | | 0.0 | | 67.8 | | 0.11 | | -0.21 | |
| 5.1.2. | 0.0 | 0.0 | 0.0 | 0.0 | 0.0 | 0.0 | | 0.0 | 7.9 | | 20.9 | | 0.0 | | 775.1 | | 803.8 | | 1.26 | | 3.18 | |
| Total | 117.5 | 5129.9 | 1764.9 | 41.7 | 41755.1 | 9344.1 | | 1458.7 | 851.7 | | 2714.9 | | 67.9 | | 779.1 | | 64025.5 | | 100.00 | |  | |
| % | 0.18 | 8.01 | 2.76 | 0.07 | 65.22 | 14.59 | | 2.28 | 1.33 | | 4.24 | | 0.11 | | 1.22 | | 100.00 | |  | |  | |
| Overall agreement (%) = 91.1; Kappa = 0.84; Strength of agreement = Almost perfect | | | | | | | | | | | | | | | | | | | | | | |
| CLC Code: 1.1.1. Continuous urban fabric; 2. Agricultural areas; 3.1.1. Low forest; 3.2. Forest plantations; 3.3.1. Natural grasslands; 3.3.2. Shrublands; 3.4.3. Sparsely vegetated areas; 3.4.5. Glaciers; 4.1.2. Peatbogs and high-Andean wetlands; 5.1.1. Water courses; 5.1.2. Water bodies. * Change between years was calculated as ((Area_i_ in 2009 – Area_i_ in 2000)/Area_i_ in 2000) x 100, where Area_i_ = area of each LCU class. | | | | | | | | | | | | | | | | | | | | | | |

Table S2: Quantification of LULC transformations (in square kilometres) and change ratio occurred between 2009 and 2013 in the study Moist Puna

| 2013 | 1.1.1. | 2. | 3.1.1. | 3.2. | 3.3.1. | 3.3.2. | 3.4.3. | 3.4.5. | 4.1.2. | 5.1.1. | 5.1.2. | Total | % | Change (%)* | |
| --- | --- | --- | --- | --- | --- | --- | --- | --- | --- | --- | --- | --- | --- | --- | --- |
| 2009 |  |  |  |  |  |  |  |  |  |  |  |  |  |  |  |
| 1.1.1. | 117.5 | 7.6 | 0.0 | 0.0 | 3.8 | 0.8 | 0.2 | 0.0 | 0.1 | 0.0 | 0.0 | 130.0 | 0.20 | 10.67 | |
| 2. | 0.0 | 5754.2 | 8.1 | 2.3 | 542.0 | 133.2 | 0.0 | 0.0 | 24.9 | 0.0 | 0.0 | 6464.8 | 10.10 | -17.69 | |
| 3.1.1. | 0.0 | 0.0 | 369.1 | 0.0 | 0.0 | 0.0 | 0.0 | 0.0 | 0.0 | 0.0 | 0.0 | 369.1 | 0.58 | -47.47 | |
| 3.2. | 0.0 | 0.0 | 0.0 | 111.5 | 10.0 | 0.0 | 0.0 | 0.0 | 0.0 | 0.0 | 0.0 | 121.5 | 0.19 | -20.66 | |
| 3.3.1. | 0.0 | 599.4 | 168.0 | 18.6 | 37457.8 | 5.7 | 0.0 | 14.3 | 1007.4 | 0.0 | 35.3 | 39306.5 | 61.39 | 0.77 | |
| 3.3.2. | 0.0 | 1492.7 | 157.4 | 20.7 | 931.3 | 10223.1 | 0.0 | 0.0 | 9.3 | 0.0 | 0.0 | 12834.5 | 20.05 | 23.85 | |
| 3.4.3. | 0.0 | 0.0 | 0.0 | 0.0 | 61.2 | 0.0 | 2022.5 | 176.5 | 21.2 | 0.0 | 2.6 | 2283.9 | 3.57 | 12.91 | |
| 3.4.5. | 0.0 | 0.0 | 0.0 | 0.0 | 0.0 | 0.0 | 0.0 | 92.1 | 0.0 | 0.0 | 0.0 | 92.1 | 0.14 | -67.44 | |
| 4.1.2. | 0.0 | 0.0 | 0.0 | 0.0 | 0.0 | 0.0 | 0.0 | 0.0 | 1573.3 | 0.0 | 0.0 | 1573.3 | 2.46 | -40.68 | |
| 5.1.1. | 0.0 | 0.0 | 0.0 | 0.0 | 0.0 | 0.0 | 0.0 | 0.0 | 0.0 | 67.8 | 0.0 | 67.8 | 0.11 | 0.00 | |
| 5.1.2. | 0.0 | 0.0 | 0.0 | 0.0 | 0.0 | 0.0 | 0.0 | 0.0 | 15.9 | 0.0 | 766.0 | 782.0 | 1.22 | -2.72 | |
| Total | 117.5 | 7853.9 | 702.7 | 153.1 | 39006.0 | 10362.8 | 2022.7 | 282.9 | 2652.3 | 67.8 | 803.8 | 64025.5 | 100.00 |  | |
| % | 0.18 | 12.27 | 1.10 | 0.24 | 60.92 | 16.19 | 3.16 | 0.44 | 4.14% | 0.11 | 1.26 | 100.00 |  |  | |
| Overall agreement (%) = 91.5 Kappa = 0.85; Strength of agreement = Almost perfect | | | | | | | | | | | | | | |  |
| CLC Code: 1.1.1. Continuous urban fabric; 2. Agricultural areas; 3.1.1. Low forest; 3.2. Forest plantations; 3.3.1. Natural grasslands; 3.3.2. Shrublands; 3.4.3. Sparsely vegetated areas; 3.4.5. Glaciers; 4.1.2. Peatbogs and high-Andean wetlands; 5.1.1. Water courses; 5.1.2. Water bodies. * Change between years was calculated as ((Area_i_ in 2013 – Area_i_ in 2009)/Area_i_ in 2009) x 100, where Area_i_ = area of each LCU class. | | | | | | | | | | | | | | |  |

Table S3: Pairwise Spearman’s rank correlation between trends of ES for the two-time periods.

| **Ecosystem service pair** | **2000-2009** | **R** | **Strength** | **2000-2013** | **R** | **Strength** | |
| --- | --- | --- | --- | --- | --- | --- | --- |
| *Regulating* |  |  |  |  |  |  | |
| Water purification and Regulation of soil erosion | 0.99 | S* | H | 0.93 | S* | H | |
| Water purification and Water flow regulation | 0.92 | S* | H | 0.93 | S* | H | |
| Water purification and Soil quality | 0.80 | S* | H | 0.96 | S* | H | |
| Water purification and Global climate regulation | 0.97 | S* | H | 0.94 | S* | H | |
| Regulation of soil erosion and Water flow regulation | 0.91 | S* | H | 0.87 | S* | H | |
| Regulation of soil erosion and Soil quality | 0.83 | S* | H | 0.91 | S* | H | |
| Regulation of soil erosion and Global climate regulation | 0.97 | S* | H | 0.86 | S* | H | |
| Water flow regulation and Soil quality | 0.58 | S* | H | 0.89 | S* | H | |
| Water flow regulation and Global climate regulation | 0.93 | S* | H | 0.94 | S* | H | |
| Soil quality and Global climate regulation | 0.79 | S* | H | 0.97 | S* | H | |
| *Regulating and Provisioning* |  |  |  |  |  |  | |
| Water purification and Crops | -0.89 | T* | H | -0.72 | T* | H | |
| Water purification and Livestock | -0.71 | T* | H | -0.52 | T* | H | |
| Regulation of soil erosion and Crops | -0.86 | T* | H | -0.86 | T* | H | |
| Regulation of soil erosion and Livestock | -0.66 | T* | H | -0.67 | T* | H | |
| Water flow regulation and Crops | -0.92 | T* | H | -0.75 | T* | H | |
| Water flow regulation and Livestock | -0.78 | T* | H | -0.63 | T* | H | |
| Soil quality and Crops | -0.51 | T* | H | -0.65 | T* | H | |
| Soil quality and Livestock | -0.28 | T | W | -0.50 | T* | H | |
| Global climate regulation and Crops | -0.83 | T* | H | -0.62 | T* | H | |
| Global climate regulation and Livestock | -0.62 | T* | H | -0.52 | T* | H | |
| *Provisioning* |  |  |  |  |  |  | |
| Crops and Livestock | 0.89 | S* | H | 0.87 | S* | H | |
| Relationship (R): synergies (S) and trade-offs (T). *Significant at a p < 0.05. Scale of correlation strength: high (H) -0.5 ≤ r ≥ 0.5, moderate (M) -0.3 ≤ r ≥ 0.3, weak (W) -0.1 < r > 0.1 | | | | | | |  |

Figure S1: Web diagrams showing the changes that provinces followed from one cluster to another during the time period: (A) Land-change dynamic clusters; (B) Bundles of ES trends; (C) Links between land-change dynamic clusters and bundles of ES trends.


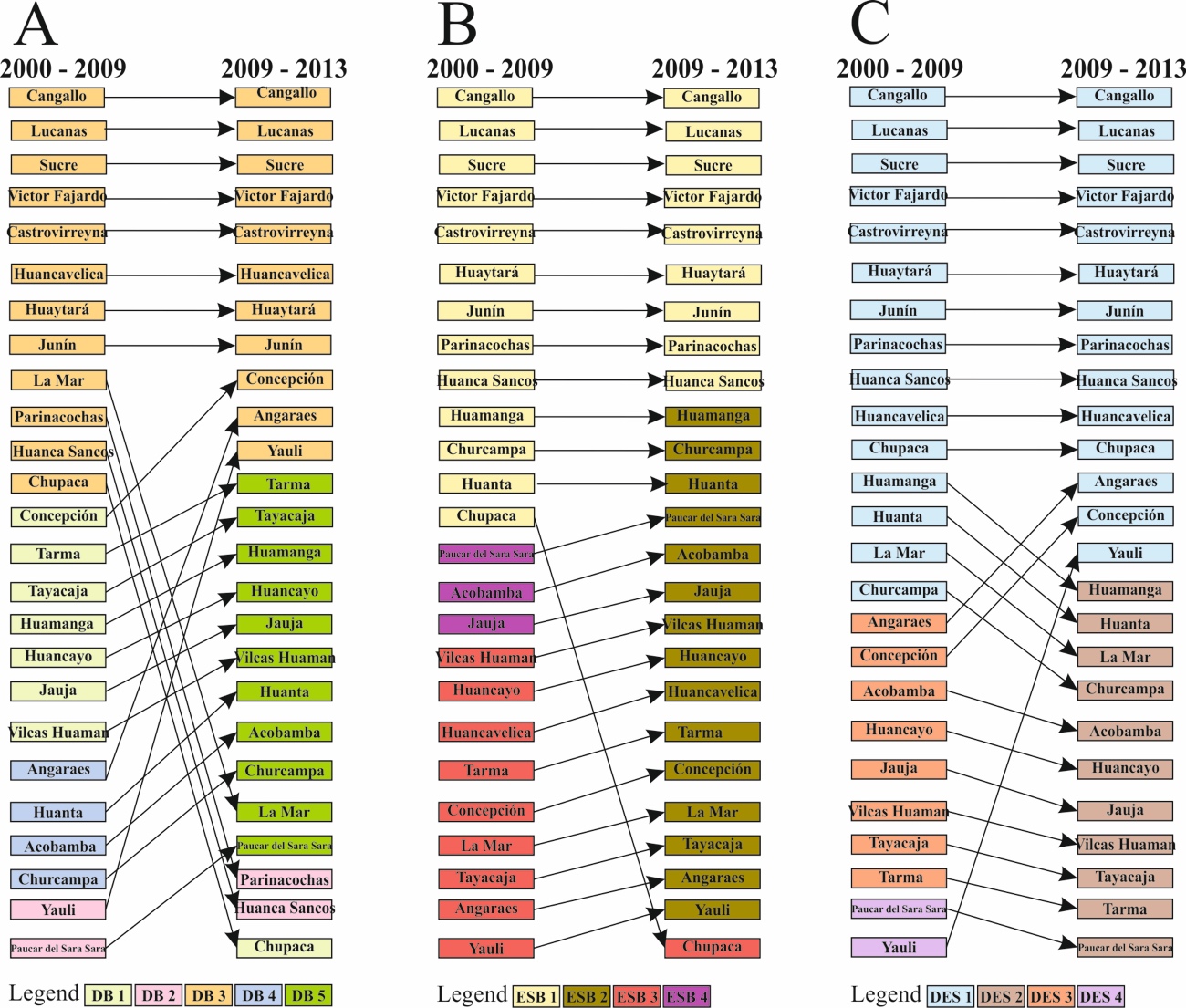


Table S4: Results of RDA analysis between land-change dynamics and ES trends for the two-time periods.

| Model T1 (2000 – 2009) | Model T2 (2009 – 2013) |
| --- | --- |
| Df AIC F Pr(>F)  - D2 1 -142.172 3.8336 0.065 .  - D3 1 -137.912 8.2607 0.010 **  - D6 1 -107.009 77.2811 0.005 **  - D1 1 -82.949 234.6823 0.005 **  - D4 0 -144.556 -Inf  ---  Signif. codes:  0 ‘***’ 0.001 ‘**’ 0.01 ‘*’ 0.05 ‘.’ 0.1 ‘ ’ 1  Inertia Proportion Rank  Total 0.042621 1.000000  Constrained 0.040470 0.949512 4  Unconstrained 0.002152 0.050488 7  Inertia is variance | Df AIC F Pr(>F)  - D6 1 -142.69 4.1895 0.055 .  - D1 1 -125.64 28.8284 0.005 **  - D2 1 -76.80 330.4457 0.005 **  ---  Signif. codes:  0 ‘***’ 0.001 ‘**’ 0.01 ‘*’ 0.05 ‘.’ 0.1 ‘ ’ 1  Inertia Proportion Rank  Total 0.048142 1.000000  Constrained 0.045874 0.952883 3  Unconstrained 0.002268 0.047117 7  Inertia is variance |

Figure S2: Spatial distribution of each driver for both time periods (distance from Lima stay invariable for both periods). The values of drivers are organised in equal interval quintiles.


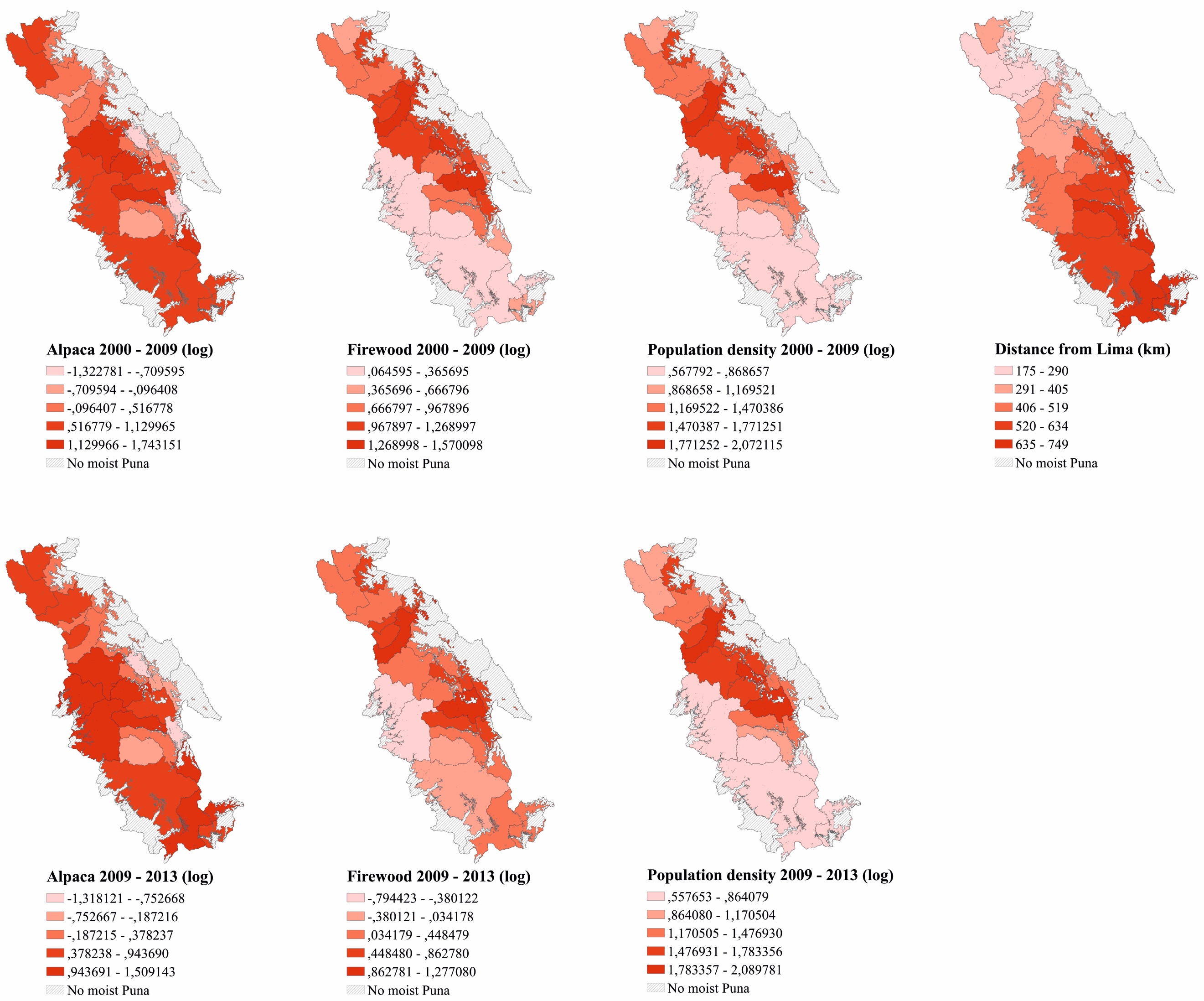


Figure S3: Redundancy analysis results across the moist Puna. The plot shows the constrain of the drivers (blue), the unconstrained dynamics and ES (red) and the association bundles (coloured points). Ecosystem service types and abbreviations: water purification (WP), regulation of soil erosion (RSE), water flow regulation (WFR), soil quality (SQ), global climate regulation (GCR), crops (CR) and livestock (LS). Dynamic types and abbreviations: agricultural expansion (D1), agricultural de-intensification (D2), deforestation (D3), urbanization (D4), afforestation (D5) and natural processes (D6). Drawings were generated with CorelDRAW X7.


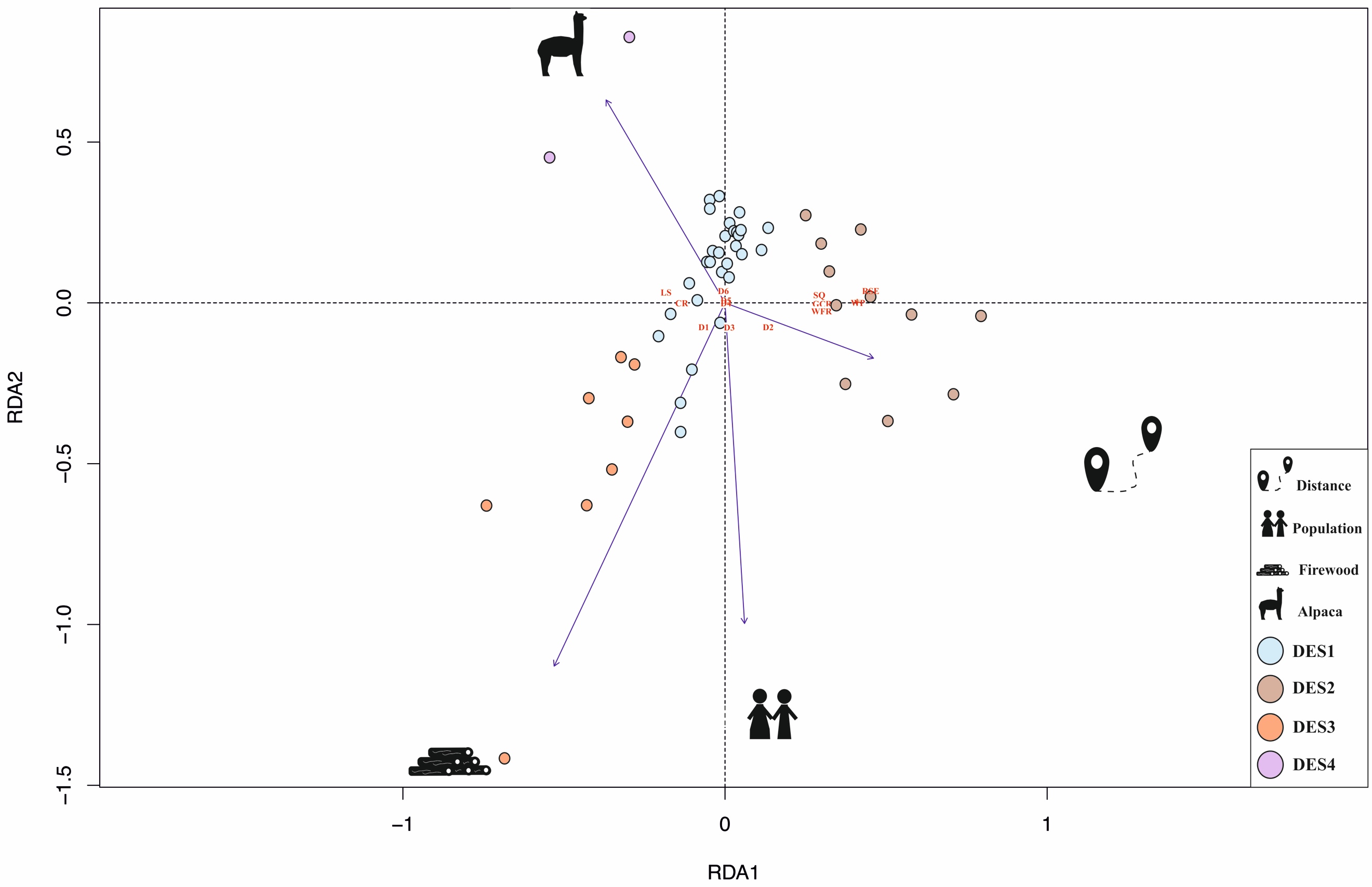


Table S5: Land cover units resulting from the features of the three time step maps

| ID | Research LULC units | Features | Data Source |
| --- | --- | --- | --- |
| 1.1.1. | Continuous urban fabric | - Cities and settlements | (1) Josse et al. (2009)^1^ |
|  |  | - Urban area | (2) Environment Ministry (2012)^2^; (3) Environment Ministry (2015)^3^ |
| 2. | Agricultural areas | - Human at work areas | (1) |
|  |  | - Crops | (2) |
|  |  | - Andean agriculture | (3) |
| 3.1.1. | Low forest | - Inter-Andean xeric montane forest and shrub lands - Low high-Andean forest - High-montane low forest and shrub lands | (1) |
|  |  | - Queñoal - Inter-Andean xeric forest | (2) |
|  |  | - Inter-Andean xeric forest - High-Andean relict forest - Meso-Andean relict forest | (3) |
| 3.2. | Forest plantation | - Human at work areas | (1) |
|  |  | - Afforestation | (2) |
|  |  | - Forest plantation (pinus and eucalyptus species) | (3) |
| 3.3.1. | Natural grassland | - High-Andean grassland - High-montane grassland | (4) INRENA (2000)^4^; (3) |
|  |  | - High-Andean grassland - Puna grass | (2) |
| 3.3.2. | Shrub lands | - Inter-Andean xeric montane shrub lands - Inter-Andean xeric shrub lands - High-montane shrub lands - High-Andean shrub lands | (1) |
|  |  | - Shrublands | (2); (3) |
| 3.4.3. | Sparsely vegetated areas | - Tundra | (4); (2) |
|  |  | - High-Andean areas with rare vegetation | (2); (3) |
| 3.4.5. | Glaciers | - Nival | (1) |
|  |  | - Glaciers | (2); (3) |
| 4.1.2. | Peatbogs and high-Andean wetlands | - High-Andean wetlands | (1); (2); (3) |
| 5.1.1. | Water courses | - Water bodies | (1) |
|  |  | - River | (2); (3) |
| 5.1.2. | Water bodies | - Water bodies | (1) |
|  |  | - Lagoons and lakes | (2); (3) |

Table S6: Respondent pool particulars

| Id | Career | Discipline | Grade | Expert affiliation | Expert Country |
| --- | --- | --- | --- | --- | --- |
| 1 | Forestry | Natural Resource Management | Master | Agricultural National University of La Molina (UNALM) | Peru |
| 2 | Biology | Ecology | Master | UNALM | Peru |
| 3 | Forestry | Natural Resource Management | Doctor | UNALM | Peru |
| 4 | Physics | Ecosystem Services | Doctor | Water Competences Centre (CCA) | Peru |
| 5 | Forestry | Ecosystem Services | Doctor | Centre for International Forestry Research (CIFOR) | France |
| 6 | Agricultural | Hydrology | Master | The mountain institute (TMI) | Peru |
| 7 | Biology | Ecology | Doctor | UNALM | Peru |
| 8 | Zootechnics | Agostology | Doctor | UNALM | Peru |
| 9 | Biology | Landscape Ecology | Master | International Potato Centre (CIP) | Peru |
| 10 | Biology | Biogeography | Doctor | Research on Arid Zones Centre (CIZA) | Peru |
| 11 | Agricultural | Water Treatment | Master | UNALM | Peru |
| 12 | Agricultural | Hydrology | Master | UNALM | Peru |
| 13 | Agricultural | Hydrology and Climate Change | Doctor | National Institute in Agricultural Innovation (INIA) | Peru |
| 14 | Biology | Natural Resource Management | Doctor | TMI | Peru |
| 15 | Biology | Environmental Sciences | Doctor | Peruvian University Cayetano Heredia (UPCH) | Peru |
| 16 | Agricultural | Hydrology | Doctor | UNALM | Peru |
| 17 | Agronomy | Soil Sciences | Doctor | UNALM | Peru |
| 18 | Biology | Agostology | Doctor | UNALM | Peru |
| 19 | Biology | Climate Change | Master | University of Toronto | Peru |
| 20 | Biology | Biogeography | Doctor | Kew Gardens | Peru |
| 21 | Biology | Natural Resource Management | Doctor | Pontifical University Catholic of Peru (PUCP) | Peru |
| 22 | Agricultural | Hydrology and Climate Change | Doctor | National Service of Meteorology and Hydrology of Peru (SENAMHI) | Peru |
| 23 | Geography | Environmental Sciences | Master | Development Andean ecoregion Consortium (CONDESAN) | Ecuador |
| 24 | Civil | Water Resources | Doctor | Independent Consultant | Peru |
| 25 | Agronomy | Natural Resources and Climate Change | Master | TMI | France |
| 26 | Geography | Biogeography | Doctor | University of Texas | USA |
| 27 | Agricultural | Hydrology | Master | National Superintendence of Sewage Services of Peru (SUNASS) | Peru |
| 28 | Agronomy | Soil Sciences | Master | Environmental Ministry of Peru (MINAM) | Peru |
| 29 | Biology | Biogeography | Master | UNALM | Peru |
| 30 | Forestry | Hydrology and Climate Change | Master | UNALM | Peru |
| 31 | Agronomy | Soil Sciences | Master | UNALM | Peru |
| 32 | Meteorology | Meteorology and Water Sciences | Master | UNALM | Peru |
| 33 | History | Biogeography and Political Ecology | Master | University of Texas | USA |
| 34 | Biology | Ecosystem Services | Graduate | Nature and Culture International (NCI) | Peru |
| 35 | Economy | Environmental Sciences | Graduate | Institute for the Promotion of Water Management (IPROGA) | Peru |
| 36 | Biology | Ecology | Master | Major National University of San Marcos (UNMSM) | Peru |
| 37 | Forestry | Natural Resource Management | Master | Research National Institute of Glaciers and Mountain Ecosystems (INAIGEM) | Peru |
| 38 | Anthropology | Natural Resources and Climate Change | Doctor | TMI | Peru |
| 39 | Biology | Ecology | Master | UNALM | Peru |
| 40 | Biology | Ecology | Master | University of Quebec | Spain |
| 41 | Civil | Hydrology | Master | Imperial College London | Ecuador |
| 42 | Civil | Hydrology | Doctor | University of Cuenca | Ecuador |
| 43 | Civil | Hydrology | Doctor | University of Cuenca | Ecuador |

Table S7: (A) Number of contributing experts for each LULC/regulating ES pairs, (B) Number of outliers, and (C) Number of experts accounted for scoring average. WP= water purification, RSE= regulation of soil erosion, WFR= water flow regulation, SQ= soil quality, GCR= global climate regulation.

|  |  |  |  | (A) |  |  |  |  | (B) |  |  |  |  | (C) |  |  |
| --- | --- | --- | --- | --- | --- | --- | --- | --- | --- | --- | --- | --- | --- | --- | --- | --- |
| CLC Code | LULC Unit | **WP** | **RSE** | **WFR** | **SQ** | **GCR** | **WP** | **RSE** | **WFR** | **SQ** | **GCR** | **WP** | **RSE** | **WFR** | **SQ** | **GCR** |
| 1.1.1. | Continuous urban fabric | 43 | 43 | 43 | 43 | 43 | 4 | 0 | 5 | 7 | 6 | 39 | 43 | 38 | 36 | 37 |
| 2. | Agricultural areas | 43 | 43 | 42 | 42 | 42 | 1 | 0 | 2 | 0 | 0 | 42 | 43 | 40 | 42 | 42 |
| 3.1.1. | Low forest | 41 | 42 | 42 | 42 | 42 | 2 | 3 | 1 | 2 | 1 | 39 | 39 | 41 | 40 | 41 |
| 3.2. | Forest plantations | 40 | 41 | 41 | 42 | 40 | 0 | 3 | 0 | 6 | 3 | 40 | 38 | 41 | 36 | 37 |
| 3.3.1. | Natural grasslands | 42 | 43 | 43 | 43 | 43 | 4 | 19 | 1 | 0 | 2 | 38 | 24 | 42 | 43 | 41 |
| 3.3.2. | Shrub lands | 41 | 41 | 41 | 41 | 41 | 3 | 2 | 1 | 1 | 3 | 38 | 39 | 40 | 40 | 38 |
| 3.4.3. | Sparsely vegetated areas | 42 | 42 | 42 | 42 | 41 | 2 | 2 | 2 | 4 | 3 | 40 | 40 | 40 | 38 | 38 |
| 3.4.5. | Glaciers | 41 | 41 | 43 | 41 | 41 | 0 | 0 | 2 | 5 | 0 | 41 | 41 | 41 | 36 | 41 |
| 4.1.2. | Peatbogs and high-Andean wetlands | 43 | 43 | 43 | 43 | 43 | 2 | 0 | 0 | 1 | 1 | 41 | 43 | 43 | 42 | 42 |
| 5.1.1. | Water courses | 40 | 41 | 41 | 41 | 39 | 0 | 0 | 0 | 0 | 0 | 40 | 41 | 41 | 41 | 39 |
| 5.1.2. | Water bodies | 42 | 42 | 43 | 42 | 42 | 7 | 0 | 4 | 0 | 0 | 35 | 42 | 39 | 42 | 42 |

Table S8: Provisioning ecosystem services assessed for the years 2000 and 2013 across the study area. Selected indicators, calculation method and source.

| Ecosystem service | Indicators | Calculation method | Source |
| --- | --- | --- | --- |
| Crops | Productive potential associated to classes within the model of main capacity of soils (scale from 0 to 3); | Sum of normalised areas (Scale from 0 to 5) with Class (A) land suitable for annual crops and class (C) land suitable for permanent crops | - Ecological Economic Zoning of Ayacucho, Regional ordinance N°003-2013-GRA/CR - Ecological Economic Zoning of Huancavelica, Regional ordinance N°257-GOB.REG-HUANCAVELICA/CR - Ecological Economic Zoning of Junín, Regional ordinance N°218-2015-GRJ/CR. |
| Livestock | Productive potential associated to classes within the model of main capacity of soils (scale from 0 to 3); | Sum of normalised areas (Scale from 0 to 5) with Class (A) land suitable for annual crops, class (C) land suitable for permanent crops and class (P) land suitable for grazing. |  |

Table S9: Details of potential drivers, proxies and units for the two-time periods (2000-2009 and 2009-2013)

| **Driver** | **Proxy measure** | **Unit** |
| --- | --- | --- |
| Population | Log average of population density for initial period.  Log average of population density for final period. | Population/km^2^ |
| Mining | Accumulated proportion of mining claims land for initial period.  Accumulated proportion of mining claims land for final period. | % |
| Alpacas | Log average of alpaca population density for initial period.  Log average of alpaca population density for final period. | Alpaca population/km^2^ |
| Goats | Log average of goat population density for initial period.  Log average of goat population density for final period. | Goat population/km^2^ |
| Firewood | Log average of population density using firewood for initial period. Log average of population density using firewood for final period. | Population using firewood/km^2^ |
| Distance from Lima | Distance from Lima | km |
| Slope | Average of slope | % |

Detailed methods for data collection of potential drivers

**Population density**

We used the log average of population density, for each time period as appropriate. This data was compiled for each year of the period. The values are estimations by the National Institute of Statistics and Informatics^5^. This data is online and publicly available.

**Mining**

Mining was calculated by taking the accumulated percentage of total land area of each province with authorised mining claims in each time period. Data were compiled for the three time steps: (1) before 2000, (2) among 2000 and 2009, and (3) between 2009 and 2013. This data is online and publicly available (Geological, Mineral and Metallurgical Institute of Peru^6^)

**Alpacas**

We used the log average of alpaca population density per square kilometre obtained by dividing the provincial alpaca population size by its area in each time period. This data was compiled for the years 1993 (III National Agriculture and Livestock Census^7^) and 2012 (IV National Agriculture and Livestock Census^8^). This data is online and publicly available.

**Goats**

We used the log average of goat population density per square kilometre obtained by dividing the provincial goat population size by its area in each time period. This data was compiled for the years 1993 (III National Agriculture and Livestock Census^7^) and 2012 (IV National Agriculture and Livestock Census^8^). This data is online and publicly available.

**Firewood**

We used the log average of population using firewood per square kilometre in each time period. This data was compiled for the period between years 2002 and 2013. The estimations are as a percentage of firewood consumption at departmental scale, produced by the National Institute of Statistics and Informatics^5^. This data is online and publicly available.

**Distance from Lima**

We calculated distance from Lima using ArcGIS and the national road network^9^ (this data is online and publicly available).

**Slope**

The average slope for each province was calculated from slope maps, classified into percentage ranks, and available in the Ecological Economic Zoning studies for Ayacucho (2013)^10^, Huancavelica (2014)^10^ and Junín (2015)^10^. This data is online and publicly available.

**References**

1. Josse, C. *et al.* *Mapa de ecosistemas de los andes del norte y centro. Bolivia,Colombia, Ecuador, Perú y Venezuela*. (2009).

2. Ministry of Environment. *Memoria descriptiva del mapa de cobertura vegetal del Peru*. (2012).

3. Ministry of Environment. Mapa nacional de cobertura vegetal. 1 (2015).

4. National Institute of Natural Resources. Mapa forestal del Perú. 1 (2000).

5. INEI - National Institute of Statistics and Informatics. National census. Available at: https://www.inei.gob.pe/estadisticas/censos/.

6. INGEMMET-Insitute of Geological Mineral and Metallurgical. Mining cadastre. Available at: http://geocatmin.ingemmet.gob.pe/geocatmin/.

7. INEI - National Institute of Statistics and Informatics. III Censo Nacional Agropecuario 1994 (III CENAGRO). Available at: http://censos.inei.gob.pe/bcoCuadros/IIIcenagro.htm.

8. INEI - National Institute of Statistics and Informatics. IV Censo Nacional Agropecuario 2012. Available at: http://censos.inei.gob.pe/Cenagro/redatam/.

9. Ministry of Transport and Communications. Road Network. Available at: https://portal.mtc.gob.pe/estadisticas/descarga.html.

10. Ministry of Environment. ZONIFICACIÓN ECOLÓGICA Y ECONÓMICA – ZEE. Available at: http://geoservidor.minam.gob.pe/zee-aprobadas/zee-aprobadas/.
